# BEEP—Bodily and Emotional Perception of Pain. A Questionnaire to Measure Reaction to Pain in Chronic Pain Disorders

**DOI:** 10.3389/fpsyg.2019.00480

**Published:** 2019-03-12

**Authors:** Antonio Preti, Serena Stocchino, Francesca Pinna, Maria Cristina Deidda, Mario Musu, Federica Sancassiani, Ferdinando Romano, Sergio Machado, Gabriele Finco, Mauro Giovanni Carta

**Affiliations:** ^1^Department of Medical Sciences and Public Health, University of Cagliari, Cagliari, Italy; ^2^Department of Public Health and Infectious Diseases, University of Rome “La Sapienza”, Rome, Italy; ^3^Instituto de Psiquiatria da Universidade Federal do Rio de Janeiro, Universidade Salgado de Oliveira (UNIVERSO), Rio de Janeiro, Brazil

**Keywords:** fibromyalgia, pain management, psychiatric comorbidity, psychological factors, psychometrics

## Abstract

**Background:** The assessment of pain and its impact on quality of life is central to the evaluation of chronic pain syndromes. However, most available tools focus on the nociceptive experience of pain, and at best only consider the occurrence of anxious, depressive, or cognitive problems. Here is a new questionnaire aimed at measuring the multifaceted impact of pain in chronic pain syndromes, the Bodily and Emotional pErception of Pain (BEEP).

**Methods:** All consecutive patients who accessed a center for the treatment of pain were invited to take part in the study. The sample included 222 participants (51 with fibromyalgia, 84 with low back pain; 87 with other chronic pain syndromes). Women were 77% of the sample, the mean age was 61 ± 15. Participants completed the BEEP, the Patient Health Questionnaire-9 (PHQ-9), and the Mood Disorder Questionnaire (MDQ).

**Results:** Reliability was good for all questionnaires. The expected three dimensions of the BEEP were confirmed by confirmatory factor analysis, and a bifactor model with three orthogonal factors showed a good fit as well. Participants diagnosed with fibromyalgia showed higher scores on the BEEP than the participants who had been diagnosed with low back pain or other chronic pain syndromes. The prevalence of probable cases of major depression and bipolar disorder in the sample was higher than expected for non-clinical samples. Levels of depression, as measured by the PHQ-9, were associated with the three dimensions of the BEEP and with the intensity of pain.

**Conclusions:** The BEEP is a promising measure of the impact of pain in daily life and differentiates fibromyalgia from other chronic pain syndromes. The BEEP may be helpful to evaluate the patient's response to the treatment over time and may favor the identification of unmet needs in patients' personal, social, and daily functioning.

## Introduction

Pain is the reaction of the body to any lesion arising from injury or illness affecting the continuity of tissues. According to the definition proposed by the International Association for the Study of Pain (IASP), pain is “An unpleasant sensory and emotional experience associated with actual or potential tissue damage, or described in terms of such damage” (Hanoch Kumar and Elavarasi, 2016). Other definitions emphasize, as well, the nociceptive component of pain (the sensory reaction to actual or potential tissue damage) and its emotional counterpart (see Hanoch Kumar and Elavarasi, 2016).

Acute pain has adaptive functions, which serve the purpose of protecting the body from further damage by attracting the attention of the subject to the harmed area(s). However, when pain persists over the long term, it loses its main function and becomes *per se* a source of suffering. In particular, chronic pain is conceived as a pain that persists beyond the normal time of healing, usually for more than 6 months (Merskey and Bogduk, 1994).

Several types of chronic pain syndromes exist, depending on the involved body areas, the hypothesized pathophysiology of the pain syndrome, and the clinical manifestations of the syndrome (Merskey and Bogduk, 1994). Many syndromes are classified among chronic pain syndromes when there is evidence that normal healing has not occurred: among others, rheumatoid arthritis, osteoarthritis, spinal stenosis, and metastatic carcinoma. Indeed, failure to heal can contribute to long-term persistence or recurrence of pain, and some of these lesions might be not detectable with current technologies.

Chronic pain syndromes have a profound impact on the quality of life of those affected by them and account for a significant proportion of the Global Burden of Disease (GBD), when estimated in terms of years lived with disability. Indeed, low back pain and migraine enlist among the first five leading causes of years lived with disability (GBD Disease and Injury Incidence and Prevalence Collaborators, 2017). Chronic pain syndromes also cause high direct and indirect costs for the patient, the family and society at large (Park et al., 2015; Driscoll and Kerns, 2016). Prevalence of chronic pain syndromes is substantial, ranging from 2 to 40% of the general population depending on the condition, its epidemiological assessment, and the characteristics of the investigated population by age, gender, and co-morbidity (van Hecke et al., 2013; Henschke et al., 2015). In Europe, it has been estimated that 2 persons out of 10 suffer from a chronic pain syndrome (van Hecke et al., 2013). Women are more likely to develop a chronic pain syndrome, with prevalence increasing with age in parallel with greater incidence of the conditions concurring to cause pain such as coronary heart disease, cerebrovascular disease, and road traffic accidents (van Hecke et al., 2013). There is also growing awareness that people with a history of abuse or violence have an enhanced risk of developing a chronic pain syndrome (Sachs-Ericsson et al., 2007; Ellsberg et al., 2008). Comorbidity with a mental disorder is very frequent, particularly with anxiety or depressive disorders (Tegethoff et al., 2015; Hooten, 2016; Pereira et al., 2017). For some syndromes, such as chronic neck or back pain or chronic headache, there is evidence that a mental disorder represents a pre-existing risk factor (Bruffaerts et al., 2015; Viana et al., 2018). In its turn, chronic pain precipitates a cascade of psychophysiological adaptations that increase, *per se*, the chance of developing anxiety and depression (Simons et al., 2014). More subtle mechanisms, still under investigation, were suggested to explain the high comorbidity of chronic pain syndromes with mental disorders (Hooten, 2016). The high co-occurrence of mental disorders with chronic pain syndromes is probably the main reason for the enhanced risk of suicide in people who had a history of chronic pain (Hooley et al., 2014; Racine, 2017). Overall, people with chronic pain were reported to be twice as likely to report suicidal behaviors or to complete suicide than the general population (Racine, 2017).

Among the chronic pain syndromes, fibromyalgia has the highest prevalence of comorbid mood disorders with an estimated 12-months prevalence of high-level depressive symptoms in up to 50% of diagnosed individuals (Carta et al., 2006; Aguglia et al., 2011; Chang et al., 2015). Fibromyalgia is characterized by chronic widespread pain and heightened and painful response to pressure in association with extreme fatigability, sleep disturbance and joint stiffness (International Classification of Diseases, Tenth Revision, Clinical Modification [ICD-10-CM], Code M79.7; American Medical Association, 2016).

A fraction of patients also experiences bowel and bladder abnormalities, numbness and tingling, and cognitive dysfunction (ICD-10-CM, Code M79.7; American Medical Association, 2016). Fibromyalgia is estimated to affect 2–8% of the general population (Clauw, 2014). Its etiology is unknown; its symptoms are thought to be a reflection of some sort of amplification of a centralized pain state, i.e., the interpretation of nociceptive stimuli by dedicated areas of the central nervous system (Clauw, 2014). Patients with fibromyalgia are likely to suffer an enhanced emotional reaction to pain as a consequence of comorbid mental disorders. This might lead to some delay in diagnosis, since fibromyalgia is sometimes interpreted within the spectrum of somatic symptoms and related disorders (Häuser and Henningsen, 2014), an approach that some authors still consider well-grounded (Tavel, 2015).

The assessment of pain and its impact on quality of life are central in the evaluation of fibromyalgia and of other chronic pain syndromes. However, most available tools focus on the nociceptive experience of pain, and at best only consider the occurrence of anxious, depressive or cognitive problems (see review in Wang et al., 2015). Among the most used measures of pain, the Brief Pain Inventory (BPI) lists only a few items concerning mood, sleep, relationship with people, which are mixed with other items aimed at measuring interference with walking capacity, working ability, and general functioning (Cleeland, 1985; Caraceni et al., 1996). The Revised Fibromyalgia Impact Questionnaire (FIQR) includes a more detailed set of items on anxiety, depression, sleep problems, memory problems, but again these are mixed with items pertaining to stiffness, energy, balance or sensitivity to noise (Bennett et al., 2009). The West Haven-Yale Multidimensional Pain Inventory (MPI), designed within a biopsychosocial framework that assumes that chronic pain arises from a dynamic interaction of biological, psychological and socio-cultural factors, comprises three sets of items. One set of items concerns the influence of pain on daily life, with items addressing the level of pain, the ability to work, mood, the satisfaction or enjoyment in social or recreational activities or with family activities, and so on; another set is aimed at measuring the responsiveness of significant others to the candidate's experience of pain; and the third set rates the frequency of common household or recreational activities (Kerns et al., 1985). These three sets of items are expected to aggregate into 12 subscales about pain severity, interference, life control, affective distress, support, negative responses, solicitous responses, distracting responses, household chores, outdoor work, activities away from home, social activities. Despite performing a multidimensional assessment, the emotional reaction to pain in the MPI is measured by 3 items only. Moreover, the MPI is long (52 items or 56 in its second, revised version) and its factorial structure proved difficult to replicate in independent studies (e.g., Deisinger et al., 2001; Andreu et al., 2006).

Our goal was to develop a measure focused on the emotional reaction to pain that would include the assessment of the limitations caused by pain in daily life, the interference caused by pain in personal and social functioning and, obviously, an estimation of the severity of pain at the time of the measurement. In our view, the emotional reaction to pain is particularly important in fibromyalgia, since it magnifies the impact of the nociception and because of the effects that a comorbid mood or anxiety disorder may have on the perception of pain via the emotional reaction to pain itself.

This paper proposes a new questionnaire to measure the multifaceted impact of pain in chronic pain syndromes. The Bodily and Emotional Perception of Pain (BEEP) assesses three dimensions: the emotional reaction to pain, the limitations caused by pain in daily life, and the interference caused by pain in personal and social functioning. Two visual analog scales complete the tool, aimed at assessing the intensity of the pain perceived in the latest 24 h and while completing the questionnaire. This article illustrates the results of the first pilot testing of the BEEP, aimed at providing preliminary evidence on its reliability, its factor structure, and its capacity of distinguishing different types of chronic pain syndromes.

## Methods

The Institutional Review Board of the University Hospital of Cagliari, Italy, approved the protocol of the study, which was conducted in accordance with the guidelines of the 1995 Declaration of Helsinki and its revisions (World Medical Association, 2013).

### Participants and Procedures

The study has been conducted at the Center for pain management and palliative medicine of the University Hospital of Cagliari, Italy. All consecutive patients who accessed the Center from January 2017 to June 2017 were invited to take part in the study. Overall, 222 agreed to participate in the study.

Patients were diagnosed according to the criteria of the ICD-10 (International Classification of Diseases and Related Health Problems−10th edition; World Health Organization, 1992) and its revision (ICD-10-CM; American Medical Association, 2016). As for fibromyalgia, the diagnostic criteria of the American College of Rheumatology were also used (Wolfe et al., 2016).

Inclusion criteria were: diagnosis of a chronic pain syndrome according to ICD-10 or ICD-10-CM; aged 18 years old or older; being able to provide informed consent. Exclusion criteria were: concomitant cancer; a current ICD-10 diagnosis of alcohol or substance dependence; pregnancy during the assessment; intellectual disability.

Patients were evaluated individually in a private area of the outpatient clinic in which they were enrolled. Participants signed the informed consent then received a booklet containing the questionnaires listed below, which they were asked to complete.

### Measures

General socio-demographic information from self-report data was collected for the following variables: age, sex, and socioeconomic status. As a measure of socioeconomic status, we used the highest level of education (Galobardes et al., 2006), which was further classified into three categories: lower than high school diploma, high school diploma, college graduate or higher.

The BEEP is a self-report questionnaire aimed at measuring the impact of chronic pain in daily life. The questionnaire was developed by a mental health rehabilitation technician with 1-year experience in the rehabilitation of people with chronic pain syndromes, a graduate in medicine and surgery with 1-year involvement in the study of psychosocial adaptation in people with chronic pain syndromes, a psychiatrist with 15 years of clinical experience with people with chronic pain syndromes, and a physician who was expert in pain management and palliative care and had over 20 years of experience in diagnosing and treating people with chronic pain syndromes.

A list of the available questionnaires measuring pain and its impact on people with chronic pain syndromes was compiled and the available tools were analyzed (see details in Wang et al., 2015). In particular, the BPI (Cleeland, 1985; Caraceni et al., 1996) and the FIQR (Bennett et al., 2009) were used as a reference. After a thorough examination of the somatic, cognitive and emotional components of pain, a series of items were created to tap into three areas of impact, also taking into account how the topics were addressed in past questionnaires (question wording, type and direction of scoring, and so on). The final version of the questionnaire was then agreed upon following a process of discussion and revision.

Cognitive debriefing with pilot testing of five patients from the target population (who were not included in this study) was arranged to identify potential issues or unclear terms. Three items with reverse scoring were deleted since they confused the participants; they were replaced with three items scored in the same direction as the others. The cognitive debriefing with patients provides the questionnaire with some degree of content validity; more formal testing of discriminant content validity (see, for example, Johnston et al., 2014) was not applied.

The BEEP is divided into two parts. The first section has 23 items rated on a six-step Likert scale (from 0 to 5) that enquire three dimensions of pain-induced reactions: the emotional reaction to pain (comprising 15 items), the limitations to daily life caused by pain (4 items), and the interference caused by pain in personal and social functioning (4 items). The Likert scale with a six-step interval was chosen to avoid a response in the middle of the scale, which is seen as a sort of “neutral” response. The interference or the limitations caused by pain were defined through less items than the emotional reaction to pain, since we expected that interference and pain-induced limitations are more easily detected and appreciated by patients than their emotional reaction to pain. Indeed, patients with fibromyalgia tend to use emotional-avoidance strategies (van Middendorp et al., 2008), while generally patients with chronic pain show a high prevalence of alexithymia and may have difficulties in identifying whether their feelings are related to pain or not (Di Tella and Castelli, 2016).

The scores on the three dimensions of the BEEP are calculated by summing the items in the same dimension, then averaging the result by the number of items in that dimension in order to compensate for the unequal number of items per dimension; finally, the score is linked back to the original Likert scale (from 0 to 5).

The second part of the BEEP includes two visual analog scales (VAS) graduated from 0 to 10, which measure the intensity of pain in the latest 24 h and at the time of questionnaire completion (the questionnaire is in the Appendix).

Since chronic pain syndromes are highly comorbid with mood disorders, the following screening tools were used to investigate the prevalence of mood disorders in the sample: the Patient Health Questionnaire-9 (PHQ-9) and the Mood Disorder Questionnaire (MDQ).

The PHQ-9 is a self-administered interview that taps into the nine DSM-IV-TR (Diagnostic and Statistical Manual of Mental Disorders-Fourth Edition; Text Revision) criteria for major depression (Kroenke et al., 2001). Each item is scored from “0” (the symptom is completely absent) to “3” (the symptom is present almost every day). The global score is the sum of the scores on each item. The threshold for major depression was set at ≥10 (Manea et al., 2012). The Italian version of the PHQ-9 was used in the study (Rizzo et al., 2000).

The MDQ is a 13-item yes/no self-report screening aimed at identifying people with probable bipolar disorder (Hirschfeld et al., 2003). Questions focus on symptoms of hypomania and mania, as described in the DSM-IV (Diagnostic and Statistical Manual of Mental Disorders-Fourth Edition) or based on clinical experience. The global score is the sum of the “yes” replies (scored as “1”) on each item. A threshold of 7 was set to identify participants with probable bipolar disorder (Weber Rouget et al., 2005). The Italian version of the MDQ was used in the study (Hardoy et al., 2005).

In Italian nationally representative samples, the frequency of probable cases of major depression and bipolar disorder–as estimated with screening tools–was 14% for major depression on the PHQ-9 in a randomized sample of 1,200 individuals (Moro et al., 2015), and 4% for bipolar disorder on the MDQ in 804 participants randomized from a community sample (*n* = 3,398; Carta et al., 2014).

### Statistical Analysis

All data were coded and analyzed using the Statistical Package for Social Sciences (SPSS) version 20. Additional analyses were carried out in R (R Core Team, 2017). All tests were two-tailed. The significance threshold was set at *p* < 0.05.

Means with standard deviations were reported for continuous variables. Counts and percentages were reported for categorical variables. Normality in the distribution of scores for the BEEP was tested with the quantile-quantile plot (qqplot), a graphical method in which the probability distribution of the data and the expected normal curve are compared by plotting the respective quantiles against each other; and with the Jarque–Bera test of normality, which tests whether data match a normal distribution by taking into account skewness and kurtosis (Jarque and Bera, 1987). ANOVA was applied to comparisons by diagnostic groups. The Games-Howell test was used to test *post-hoc* differences by groups, since it does not assume equal variances and sample size. The effect size of the differences was calculated on the basis of partial η^2^, which is the fraction of the variance in the scores that can be attributed to the independent factor.

Scales reliability was measured by Cronbach's alpha. For group comparisons, reliability values of 0.70 are considered satisfactory (Kottner et al., 2011), and when dealing with subscales derived from a single questionnaire, values around 0.60 are considered acceptable (Nunnally and Bernstein, 1994).

The distribution of the first part of the BEEP into the a-priori expected three dimensions was tested with confirmatory factor analysis (CFA). CFA was carried out with the *lavaan* package running in R (Rosseel, 2012) and was conducted with a maximum likelihood estimation with robust standard errors and a Satorra-Bentler scaled test statistic, to correct for the ordinal nature of the Likert scales and the manifest violation of the multivariate normality assumption (Mardia test: skew = 3,614, *p* < 0.0001; kurtosis = 14.55, *p* < 0.0001). The fit of the models was evaluated on the basis of usual indices, such as the comparative fit index (CFI), the root mean square error of approximation (RMSEA), and the standardized root mean square residual (SRMR). RMSEA values of 0.08 or less, SRMR of 0.09 o lower and CFI equal to 0.90 or higher are conventionally accepted as evidence of good fit of the model (Browne and Cudeck, 1993; Hu and Bentler, 1999). McDonald's omega was also calculated, as estimated by the model (McDonald, 1978). McDonald's omega is a coefficient of reliability, with the advantage of taking into account the strength of the association between elements and constructs, as well as measurement errors specific to each element. McDonald's omega ≥0.90 is considered optimal.

Three models were tested: a unidimensional model, which assumes that all items tap into a single dimension of reaction to pain; the a-priori expected three-factor model; and a hierarchical model with the a-priori expected three factors correlating with each other and converging into a second-order factor of reaction to pain. The reliability of the second-order factor was calculated as McDonald's omega. We reported both the proportion of the total variance of the first-order factors explained by the presence of the second-order factor; and the partial coefficient omega, i.e., the variance proportion of the observed scores due to the second-order factor after taking into account the effect of the first-order factors.

As an alternative model we also tested a bifactor implementation of the three-factor model. The bifactor model with three nuance dimensions was applied to test a general factor resulting from the loading of all 23 items on a single dimension of “reaction to pain.” Additional residual variance was explained by the items pertaining to each scale and loaded on their proper scale, which resulted in three orthogonally independent factors.

To check for reasonable unidimensionality of the general factor extracted from the bifactor models, the explained common variance (ECV), the percentage of uncontaminated correlations (PUC), and Omega Hierarchical were calculated (see Rodriguez et al., 2016). ECV was calculated as the ratio of the variance explained by the general factor to the variance explained by the model (i.e., the variance explained by the general factor plus the variance explained by the group factors, i.e., the three orthogonally independent factors). The PUC is the ratio of the number of uncontaminated correlations to the number of unique correlations and was calculated by taking into account the number of items for each group factor. The Omega Hierarchical reflects the percentage of the systematic variance in unit-weighted total scores that can be attributed to the individual differences in the general factor. All these indexes range from 0 to 1. As a rule, ECV is expected to be higher than 0.70; however, with PUC >0.80, general ECV values are less important in predicting bias; when PUC <0.80, ECV higher than 0.60 and Omega Hierarchical higher than 0.70 provide evidence that some multidimensionality is not severe enough to disqualify the interpretation of the instrument as primarily unidimensional (Rodriguez et al., 2016).

The Relative Omega was computed, too, i.e., the ratio of Omega Hierarchical to the Omega, which is the model-based estimate of internal reliability of the multidimensional structure. For the general factor, the Relative Omega is the percentage of the reliable variance in the multidimensional structure that is attributable to the general factor itself; for specific factors, it represents the proportion of reliable variance in the subscale that is independent from the general factor (details in Rodriguez et al., 2016).

The ECV, the PUC, the Omega, the Omega Hierarchical, and the Relative Omega were calculated with a Microsoft Excel-based calculator developed by Dueber (2016).

The models were compared on the basis of the goodness-of-fit indexes and the Bayesian information criterion (BIC, Schwarz, 1978) and its derivation, the Sample Size Adjusted BIC (ssBIC, Sclove, 1987). The models with the lowest BIC and ssBIC should be preferred (Kim et al., 2015).

The capacity of the BEEP to discriminate patients with fibromyalgia from patients with other chronic pain syndromes was evaluated via ANOVA (with Games-Howell *post-hoc* test) and the receiver operating characteristic (ROC) curve analysis. Results of the ROC analysis were summarized by reporting: the area under receiver operator characteristic curve (AUC), with 95% confidence interval (CI); sensitivity, specificity, positive and negative predictive value, and the positive diagnostic likelihood ratio. Conventional thresholds for AUC are 0.80 to 0.90, good; 0.70 to 0.80, fair; <0.70, poor.

The ROC curves were compared with the method of DeLong et al. (1988). ROC analysis was conducted with the pROC package running in R (Robin et al., 2011).

The impact of affective disorders on the three dimensions of the BEEP was investigated by assessing the associations of the scores on the PHQ-9 and the MDQ with the scores on the BEEP by using Pearson's correlation coefficient. To further explore the impact of an affective disorder on the BEEP scores, we tested an interaction model of affective disorder caseness and chronic pain syndrome diagnosis. This analysis was carried out with the package *jtools* running in R (Long, 2018).

## Results

The sample included 222 participants, of whom 51 with a diagnosis of fibromyalgia, 84 diagnosed with low back pain, 61 diagnosed with arthritis or similar syndromes, plus a minority of neuropathies (*n* = 13), rheumatoid arthritis (*n* = 9), and tendinopathy (*n* = 3), grouped under the heading “other chronic pain syndromes.”

Table 1 summarizes the main characteristics of the sample.

**Table 1:** General socio-demographic and clinical characteristics of the sample (*n* = 222).

	**Fibromyalgia**	**Low back pain**	**Other chronic pain syndromes**	
	***N*** **(%)**	***N*** **(%)**	***N*** **(%)**	
	**51 (23%)**	**84 (38%)**	**87 (39%)**	**Statistics**
**GENDER**				
Men	4 (8%)	27 (32%)	21 (24%)	χ^2^ = 10.4, df = 2, *p =* 0.005
Women	47 (92%)	57 (68)	66 (76%)	
**AGE**				
Mean (*SD*)	48 (12)	64 (15)	65 (12)	*F* = 30.7, df = 2;219, *p* < 0.0001
**EDUCATION**				
Compulsory school	16 (31%)	40 (48%)	30 (34%)	χ^2^ = 24.9, df = 6, *p* < 0.0001
High school or equivalent	25 (49%)	13 (15%)	19 (22%)	
University degree or higher	4 (8%)	9 (11%)	7 (8%)	
Missing information	6 (12%)	22 (26%)	31 (36%)	
**PHQ-9**				
Cronbach's α (95%CI)	0.73 (0.58–0.85)	0.75 (0.65–0.82)	0.81 (0.69–0.86)	
Mean (*SD*)	14.2 (5.0)	9.5 (4.9)	10.1 (5.5)	*F* = 10.1, df = 2;183, *p* < 0.0001
Possible case of major depression	28 (82%)	36 (49%)	39 (49%)	χ^2^ = 12.2, df = 2, *p =* 0.002
**MDQ**				
Cronbach's α	0.84 (0.74–0.91)	0.82 (0.76–0.88)	0.80 (0.74–0.88)	
Mean (*SD*)	3.9 (3.4)	2.9 (2.9)	3.2 (2.8)	*F* = 1.26, df = 2;183, *p =* 0.28
Possible case of bipolar disorder	5 (15%)	5 (7%)	8 (10%)	χ^2^ = 1.80, df = 2, *p =* 0.40
Missing clinical information	17 (33%)	11 (13%)	8 (9%)	χ^2^ = 14.7, df = 2, *p =* 0.001

The sample included a majority of women, especially in the group diagnosed with fibromyalgia. The age range of the sample was wide, from 22 to 89 years. Participants diagnosed with fibromyalgia reported a younger age than participants with a diagnosis of low back pain or other chronic pain syndromes. Participants diagnosed with fibromyalgia also had a higher education level than the other two groups; they were also more willing to communicate their education level than the other groups of participants.

Some patients did not complete the additional psychopathology measures (i.e., PHQ-9 and MDQ). Missing information was more frequent in the participants diagnosed with fibromyalgia than in the other two groups (see details in Table 1).

Among those who completed the screening tools on depression and bipolar disorder, the reliability of PHQ-9 and MDQ–measured as Cronbach's α-was optimal with values above the conventional threshold of 0.70. Among those who completed the questionnaire, participants diagnosed with fibromyalgia reported higher scores on the PHQ-9 than those in the other two groups (*p* < 0.001 on the Games-Howell *post-hoc* test on both comparisons; partial η^2^ = 0.184). No statistically significant differences were observed on the MDQ (partial η^2^ = 0.014). See details in Table 1.

Overall when compared with data from Italian nationally representative samples, the prevalence of cases of probable major depression and probable bipolar disorder in the sample was higher than in non-clinical samples (probable major depression on the PHQ-9: χ^2^ = 194.52, df = 3, *p* < 0.0001; probable bipolar disorder on the MDQ: χ^2^ = 13.27, df = 3, *p* = 0.0041).

### Confirmatory Factor Analysis of the Bodily and Emotional Perception of Pain

The three-factor model had a better fit and lower BIC and ssBIC values than the unidimensional model. The hierarchical, second-order model was indistinguishable from the three-factor model. Although the CFI was not optimal after “robust” correction, both the three-factor model and its hierarchical implementation can be considered acceptable on the basis of all the other indexes (Table 2).

**Table 2:** Goodness-of-fit indices of the tested models (*n* = 222).

**Model**	**χ^**2**^**	**df**	**CFI**	**Robust CFI**	**RMSEA (95%CI)**	**SRMR**	**McDonald ‘s omega**	**BIC**	**ssBIC**
One-factor	428.9, *p* < 0.0001	230	0.893	0.866	0.071 (0.061–0.082)	0.062	0.92	18,293	18,074
Three-factor	382.1, *p* < 0.0001	227	0.916	0.897	0.063 (0.052–0.074)	0.058	0.92	18,247	18,019
Three-factor with second order factor	382.1, *p* < 0.0001	227	0.916	0.897	0.063 (0.052–0.074)	0.058	0.92	18,247	18,019
Bifactor implementation of the three-factor model	329.92, *p* < 0.0001	207	0.934	0.918	0.059 (0.047–0.070)	0.053	0.92	18,290	17,998
Threshold for fit	*p* > 0.05		>0.90	>0.90	<0.08	<0.09	≥0.90		

The bifactor implementation of the three-factor model had a modestly better fit than the three-factor model and its hierarchical implementation (see Table 2).

The ECV of the general factor of the bifactor model with three orthogonal factors was 0.81, the PUC in this bifactor model was 0.54, and the Omega Hierarchical of the general factor in this bifactor model was 0.91. For this model, the Omega was quite good for the general factor (0.93), as was its Relative Omega (0.98). Relative Omega was null for the “emotional reaction to pain” dimension (0.001), 0.26 for the “limitations caused by pain in daily life” dimension, and 0.19 for the “interference with personal and social functioning” dimension.

Overall, a bifactor implementation of the expected a-priori three-factor model is plausible, with a general factor measuring global “reaction to pain” and an additional, limited variance explained by “limitations caused by pain in daily life” and “interference with personal and social functioning caused by pain.” However, since the three-factor model fits the data reasonably well, and it is coherent with the a-priori conceptualization of the scale, we decided to give preference to it.

In the three-factor model, factor loading of the items on their factor was above 0.500 for all items but two, confirming a good fit of the model (Table 3).

**Table 3:** Factor loading of the bodily and emotional perception of pain in the three-factor model.

**Item**	**Standardized factor loading**	p^*^
**EMOTIONAL REACTION TO PAIN**		
1. Irritability	0.516	0.0001
2. Helplessness	0.565	0.0001
3. Deep sadness	0.646	0.0001
4. Sense of unfairness	0.517	0.0001
5. Pessimism	0.698	0.0001
6. Anxiety	0.508	0.0001
7. Sense of guilt	0.553	0.0001
8. Frustration	0.670	0.0001
9. Mistrust in one's own skills	0.631	0.0001
10. Fear of Incurability	0.580	0.0001
11. Confusion	0.591	0.0001
12. I do not recognize myself	0.683	0.0001
13. I feel aged	0.658	0.0001
14. I feel impaired	0.653	0.0001
15. I don't feel independent	0.550	0.0001
Estimated reliability	α = 0.894; ω = 0.895; ω^h^ = 0.894	
**LIMITATIONS CAUSED BY PAIN IN DAILY LIFE**		
16. Limited in working activities	0.600	0.0001
17. Limited in the ability to move	0.654	0.0001
18. Limited in the social role	0.737	0.0001
19. Limited in sport or leisure activities	0.393	0.0001
Estimated reliability	α = 0.666; ω = 0.675; ω^h^ = 0.673	
**INTERFERENCE WITH PERSONAL AND SOCIAL FUNCTIONING**		
20. Interference with mood	0.739	0.0001
21. Interference with social relationships	0.706	0.0001
22. Interference with sleep	0.477	0.0001
23. Interference with the pleasure of living	0.726	0.0001
Estimated reliability	α = 0.758; ω = 0.757; ω^h^ = 0.748	

*Estimated reliability for each factor was reported as Cronbach's ω, McDonald's ω, and ω_3_, which is equivalent to ω hierarchical (ω^h^)*.

* *p-value of the fit of the item on its factor. Null hypothesis (p > 0.05) is that the item does not fit on its factor*.

Latent correlations between the dimensions were high: “emotional reaction to pain” with “limitations caused by pain in daily life”: 0.78 (s.e. = 0.06), *z* = 11.8, *p* < 0.0001; “emotional reaction to pain” with “interference with personal and social functioning”: 0.87 (0.04), *z* = 20.8, *p* < 0.0001; “limitations caused by pain in daily life” with “interference with personal and social functioning”: 0.90 (0.04), *z* = 23.5, *p* < 0.0001.

Estimated reliability was optimal for the “emotional reaction to pain” and the “interference with personal and social functioning” subscales, and it was acceptable for the “limitations caused by pain in daily life” subscale (see details in Table 3).

Global reliability was optimal for the total Likert scale (Cronbach's α = 0.92; 95%CI = 0.91–0.94). In the hierarchical implementation of the three-factor model, the second-order factor showed omega = 0.95 and partial omega = 0.91.

### Descriptive Statistics

In the whole sample, scores on the three subscales were 2.39 (standard deviation [*SD*] = 1.20) for the “emotional reaction to pain” subscale, 2.58 (1.38) for the “limitations caused by pain in daily life” subscale, and 2.92 (1.29) for the “interference with personal and social functioning” subscale. Skewness was low in all subscales (respectively, −0.03, −0.38, and −0.07), and kurtosis was equally acceptably low (respectively, −0.95, −0.67, and −1.03).

Scores on the two VAS measuring the intensity of pain were 6.30 (2.88) for the intensity of pain in the last 24 h and 4.75 (3.29) for the intensity of pain at the time of completion. Again, skewness was low for both VAS (respectively, −0.52 and −0.09), while kurtosis was low for the intensity of pain in the last 24 h and modestly high for the intensity of pain at the time of completion (−0.68 and −1.36).

**Figure 1:**
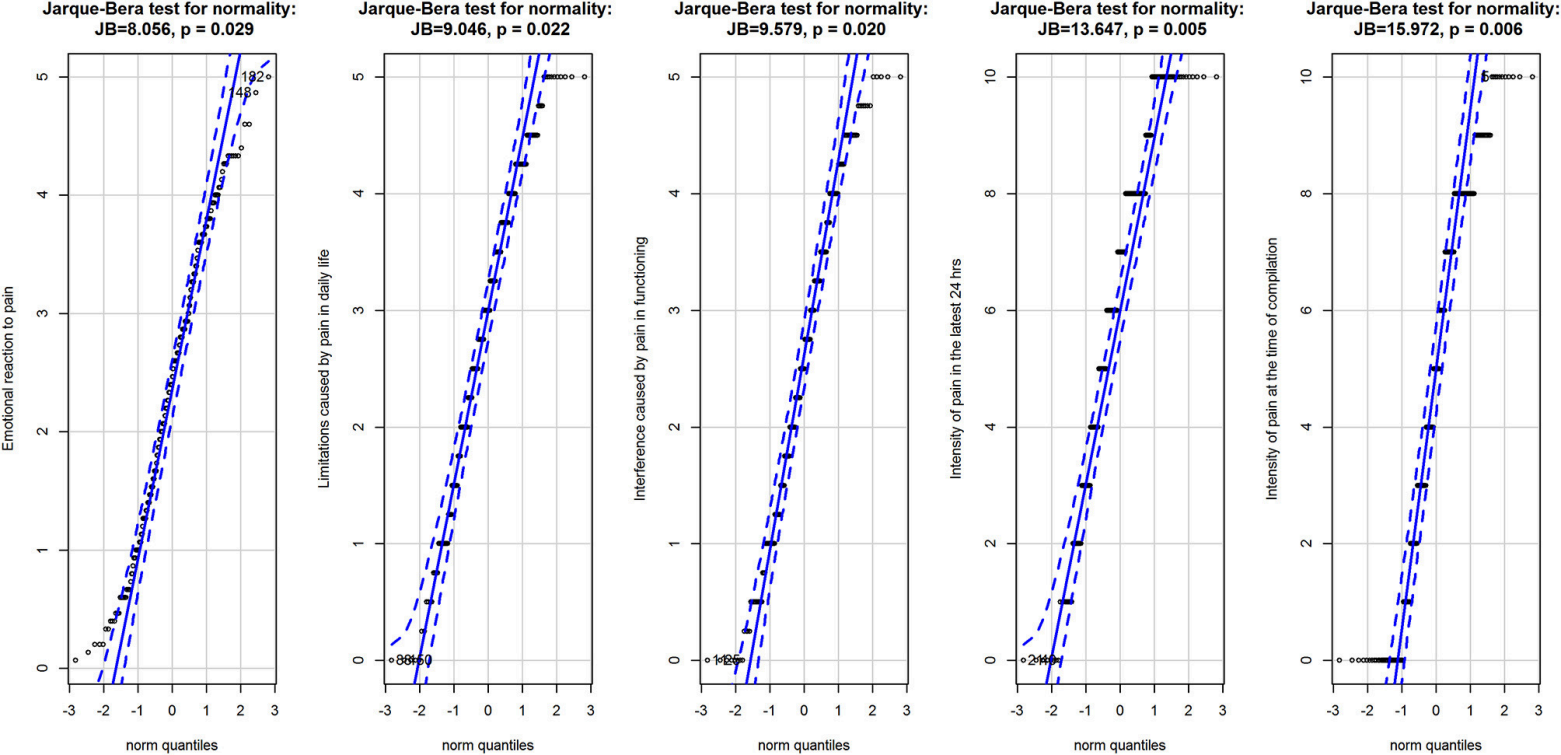
Q-Q plots for the three subscales of the BEEP and the two VAS measuring the intensity of pain. Data are represented as points with 95% confidence interval of the fit of the quantile in the data (vertical axis) against the quantile if the data were from the normal distribution (horizontal axis). Data are from the normal population when they are entirely within the 95% confidence interval of the fit. Above the graphics are the results of the Jarque-Brera test for normality for each variable.

Overall, the three subscales of the BEEP did not deviate largely from a normal distribution (Figure 1), albeit the Jarque–Bera test had *p* < 0.05. The two VAS had a more appreciable deviation from normality, as also indicated by the result of the Jarque–Bera test (see Figure 1).

### Discriminative Ability of the Bodily and Emotional Perception of Pain

In all three subscales, participants diagnosed with fibromyalgia showed higher scores than those reported by participants who had been diagnosed with low back pain or other chronic pain syndromes (Table 4).

**Table 4:** Comparison of the three groups on the dimensions of the bodily and emotional perception of pain.

	**Fibromyalgia**	**Low back pain**	**Other chronic pain syndromes**
	*N* = 51 (23%)	*N* = 84 (38%)	*N* = 87 (39%)
Emotional reaction to pain	3.26 (0.96)^a^	2.03 (1.12)^b^	2.21 (1.15)^b^
Limitations caused by pain in daily life	3.66 (1.02)^a^	2.55 (1.42)^b^	2.80 (1.16)^b^
Interference of pain with personal and social functioning	3.47 (1.09)^a^	2.14 (1.40)^b^	2.35 (1.30)^b^
ANOVA	*F*_(2, 205)_ = 20.58; *p* < 0.0001; partial η^2^ = 0.167	*F*_(2, 205)_ = 12.76; *p* < 0.0001; partial η^2^ = 0.110	*F*_(2, 205)_ = 17.06; *p* < 0.0001; partial η^2^ = 0.142

a,b *Across the diagnostic groups, those with different superscript letter differ from each other at the Games-Howell post-hoc test with p < 0.0001*.

Participants diagnosed with fibromyalgia differed from the other two groups at a statistically significant level in all three subscales (Games-Howell *post-hoc* test: *p* < 0.0001 in both comparisons). Effect size, on the basis of partial η^2^, suggested that 11 to 16% of the variance in the scores was explained by diagnostic group membership.

**Figure 2:**
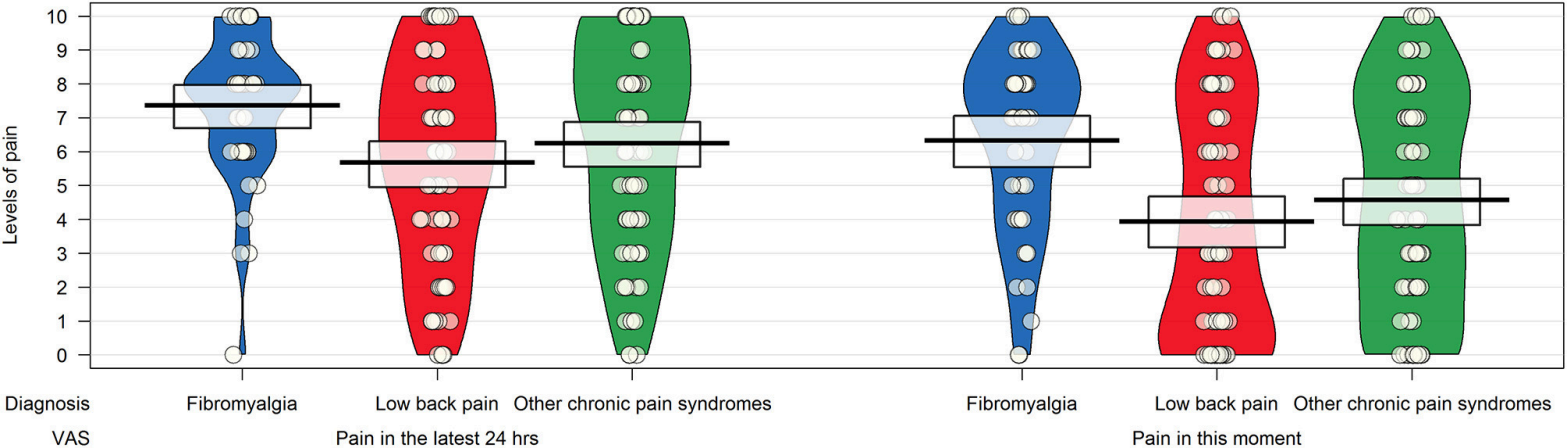
Pirateplot of the intensity of pain in patients with chronic pain syndrome as measured by items 24 and 25 of the Bodily and Emotional Perception of Pain. The pirateplot reports the raw data as points (with noise [jitter] added horizontally to reduce overlap among points with similar value); a vertical bar shows the central tendency in the data within a rectangle, with its extremes indicating the 95% confidence interval of the mean. A smoothed density of the distribution of the data is also depicted.

Participants diagnosed with fibromyalgia had higher scores on the visual analog scales measuring the intensity of pain in the last 24 h and at the time of completion, but the differences were statistically significant (*p* < 0.0001) in the group with low back pain only (Figure 2).

ROC analysis revealed that all three dimensions of the BEEP were able to differentiate patients with fibromyalgia from those with low back pain with a reasonably fair AUC (95% CI between 0.70 and 0.80; Table 5, section A, for details).

**Table 5:** Discriminative capacity of the three dimensions of the BEEP based on ROC analysis.

**ROC analysis**	**Emotional reaction to pain**	**Limitations caused by pain in daily life**	**Interference of pain with personal and social functioning**
**A. COMPARISON OF PATIENTS DIAGNOSED WITH FIBROMYALGIA AND PATIENTS DIAGNOSED WITH LOW BACK PAIN**			
AUC (95%CI)	0.796 (0.716–0.876)	0.738 (0.649–0.827)	0.760 (0.678–0.843)
Cutoff	2.8	3.5	2.7
Sensitivity	0.79	0.73	0.79
Specificity	0.72	0.68	0.64
Positive predictive value	0.65	0.60	0.59
Negative predictive value	0.84	0.80	0.83
Diagnostic likelihood ratio	2.88	2.32	2.24
**B.COMPARISON OF PATIENTS DIAGNOSED WITH FIBROMYALGIA AND PATIENTS DIAGNOSED WITH OTHER CHRONIC PAIN SYNDROMES**			
AUC (95%CI)	0.764 (0.679–0.849)	0.724 (0.632–0.815)	0.738 (0.653–0.824)
Cutoff	2.8	3.5	2.2
Sensitivity	0.79	0.73	0.89
Specificity	0.70	0.68	0.45
Positive predictive value	0.61	0.58	0.49
Negative predictive value	0.85	0.81	0.88
Diagnostic likelihood ratio	2.64	2.34	1.65

**Figure 3:**
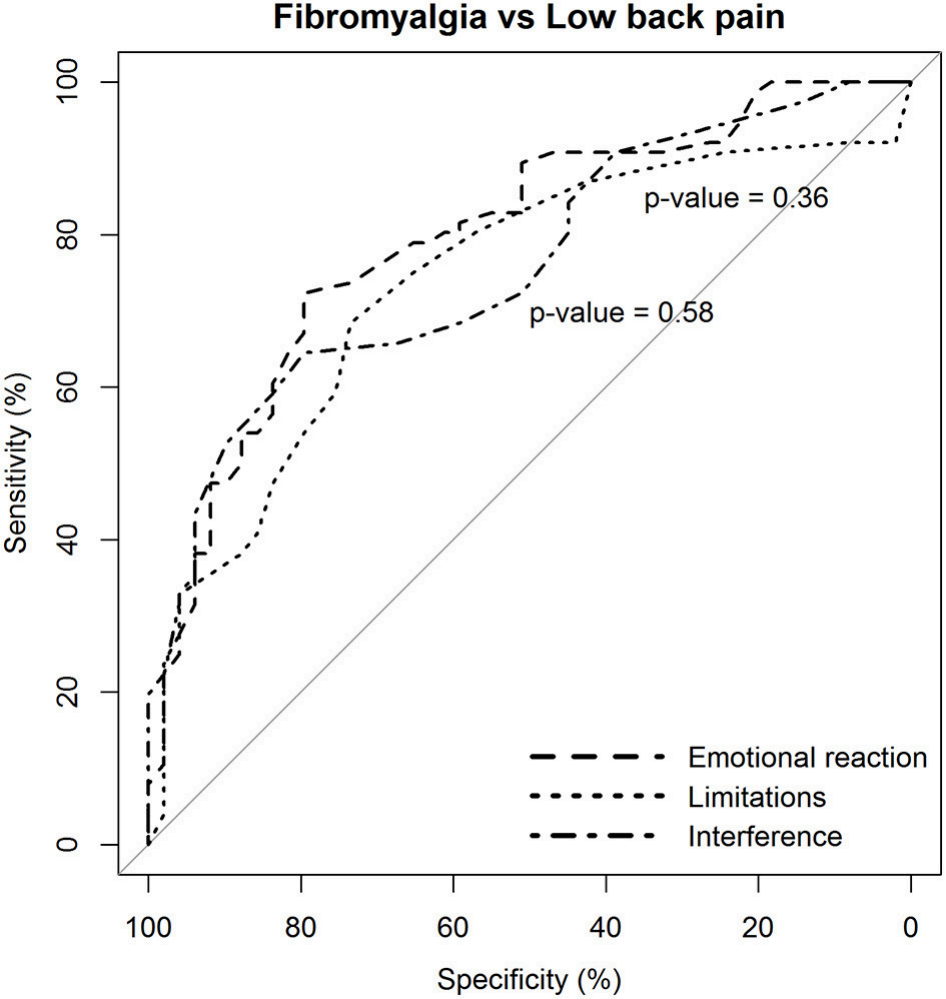
Statistical comparison of the ROC curves between the predictive capacity of the “emotional reaction to pain” subscale of the BEEP and the other two subscales–“limitations caused by pain in daily life” and “interference with personal and social functioning”–in differentiating patients with fibromyalgia from patients with low back pain. The statistical significance of the difference between the areas under the ROC curves derived from the same cases is based on the method by DeLong et al. (1988).

The two dimensions of limitations and interference caused by pain were not more likely to discriminate patients with fibromyalgia from those with low back pain than the dimension of emotional reaction to pain (Figure 3).

The diagnostic likelihood ratio was similar among the three dimensions of the BEEP. Overall, those who scored above the suggested cutoff were two times more likely to have fibromyalgia than low back pain.

Similar findings were observed in the comparison between patients with fibromyalgia and those with other chronic pain syndromes (Table 5, section B).

**Figure 4:**
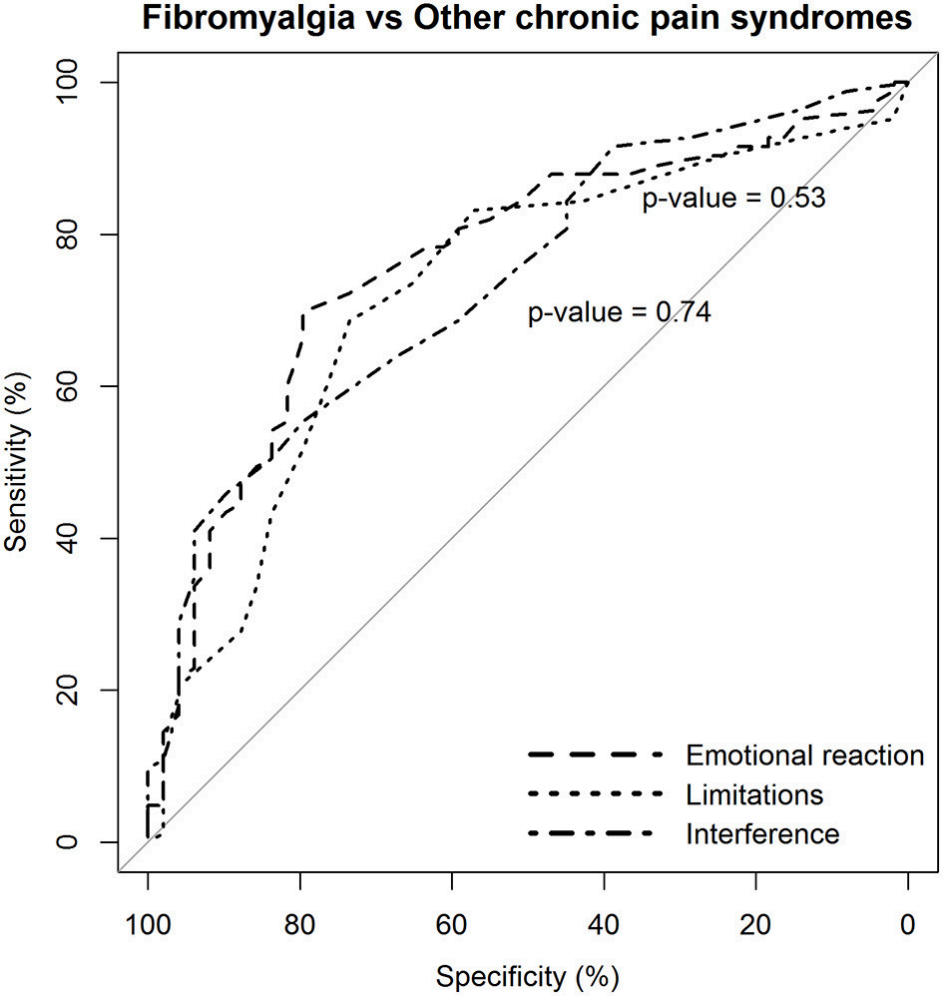
Statistical comparison of the ROC curves between the predictive capacity of the “emotional reaction to pain” subscale of the BEEP and the other two subscales–“limitations caused by pain in daily life” and “interference with personal and social functioning”–in differentiating patients with fibromyalgia from patients with other chronic pain syndromes, but excluding low back pain. The statistical significance of the difference between the areas under the ROC curves derived from the same cases is based on the method by DeLong et al. (1988).

Again, the two dimensions of limitations and interference caused by pain were not more likely to discriminate patients with fibromyalgia from those with other chronic pain syndromes than the dimension of emotional reaction to pain (Figure 4).

However, the diagnostic likelihood ratio of the “interference with personal and social functioning” subscale was lower than the diagnostic likelihood ratio of the other two subscales when discriminating the patients with fibromyalgia from those with other chronic pain syndromes. Overall, the negative predictive value of the BEEP in this enriched sample of patients with chronic pain syndromes was pretty high (≥80%), suggesting that those scoring on the BEEP below the suggested cutoff are unlikely to have fibromyalgia.

### Association of the Bodily and Emotional Perception of Pain With psychopathology

As expected, the three components of the BEEP were correlated with each other and positively associated with the intensity of pain (Table 6).

**Table 6:** Correlations between dimensions of the Bodily and Emotional Perception of Pain and measures of psychopathology.

	**1**	**2**	**3**	**4**	**5**	**6**
1. Emotional reaction to pain						
2. Limitations caused by pain in daily life	0.600^*^					
3. Interference of pain with personal and social functioning	0.693^*^	0.659^*^				
4. Intensity of pain in the last 24 h	0.416^*^	0.418^*^	0.445^*^			
5. Intensity of pain at the time of compilation	0.428^*^	0.385^*^	0.497^*^	0.750^*^		
6. PHQ-9	0.588^*^	0.475^*^	0.667^*^	0.311^*^	0.399^*^	
7. MDQ	0.303^*^	0.256	0.216	0.120	0.181	0.264^*^

* *Pearson's correlation coefficient p < 0.0001*.

Levels of depression, as measured by the PHQ-9, were associated with the three dimensions of the BEEP and with the intensity of pain. The role of bipolar predisposition, as measured by the MDQ, appears to be less important, albeit not absent, in influencing the degree of emotional or bodily reaction caused by pain.

Being a case of depression at the PHQ-9 had a statistically significant impact on the scores on the three dimensions of the BEEP (Table 7).

**Table 7:** Interaction between depression and the three BEEP dimensions of emotional reaction to pain, limitations caused by pain in daily life, and interference of pain with personal and social functioning.

**Beta (s.e. of beta)**	**Emotional reaction to pain**	**Limitations caused by pain in daily life**	**Interference of pain with personal and social functioning**
Case of depression on the PHQ-9	beta = 1.23 (0.16); *t =* 7.8; *p* < 0.0001	beta = 2.19 (0.17); *t =* 7.3; *p* < 0.0001	beta = 1.75 (0.16); *t =* 10.7; *p* < 0.0001
	*R*^2^ = 26%; Adj. *R*^2^ = 26%	*R*^2^ = 24%; Adj. *R*^2^ = 23%	*R*^2^ = 40%; Adj. *R*^2^ = 40%
Case of depression on the PHQ-9	beta = 1.18 (0.46); *t =* 2.6; *p* = 0.01	beta = 0.07 (0.46); *t =* 0.1; *p* = 0.88	beta = 1.56 (0.48); *t =* 3.2; *p* < 0.0001
Diagnosis: Low back pain	beta = −0.64 (0.45); *t =* −1.4; *p =* 0.15	beta = −1.47 (0.48); *t =* −3.0; *p* < 0.0001	beta = −0.52 (0.48); *t =* −1.1; *p* = 0.28
Diagnosis: Other pain syndromes	beta = −0.47 (0.44); *t =* −1.1; *p =* 0.29	beta = −1.41 (0.48); *t =* −2.9; *p* < 0.0001	beta = −0.59 (0.47); *t =* −1.2; *p* = 0.21
Low back pain * Depression	beta = 0.04 (0.52); *t =* 0.1; *p =* 0.94	beta = 1.16 (0.56); *t =* 2.1; *p =* 0.04	beta = −0.05 (0.55); *t =* −0.1; *p =* 0.93
Other pain syndromes * Depression	beta = −0.16 (0.51); *t =* −0.3; *p =* 0.75	beta = 1.21 (0.55); *t =* 1.2; *p =* 0.03	beta = 0.21 (0.54); *t =* −0.4; *p =* 0.70
	*R*^2^ = 30%; Adj. *R*^2^ = 28%	*R*^2^ = 28%; Adj. *R*^2^ = 26%	*R*^2^ = 41%; Adj. *R*^2^ = 40%

This was particularly evident for the subscale “interference of pain with personal and social functioning,” with up to 40% of variance explained by suffering from depression.

**Figure 5:**
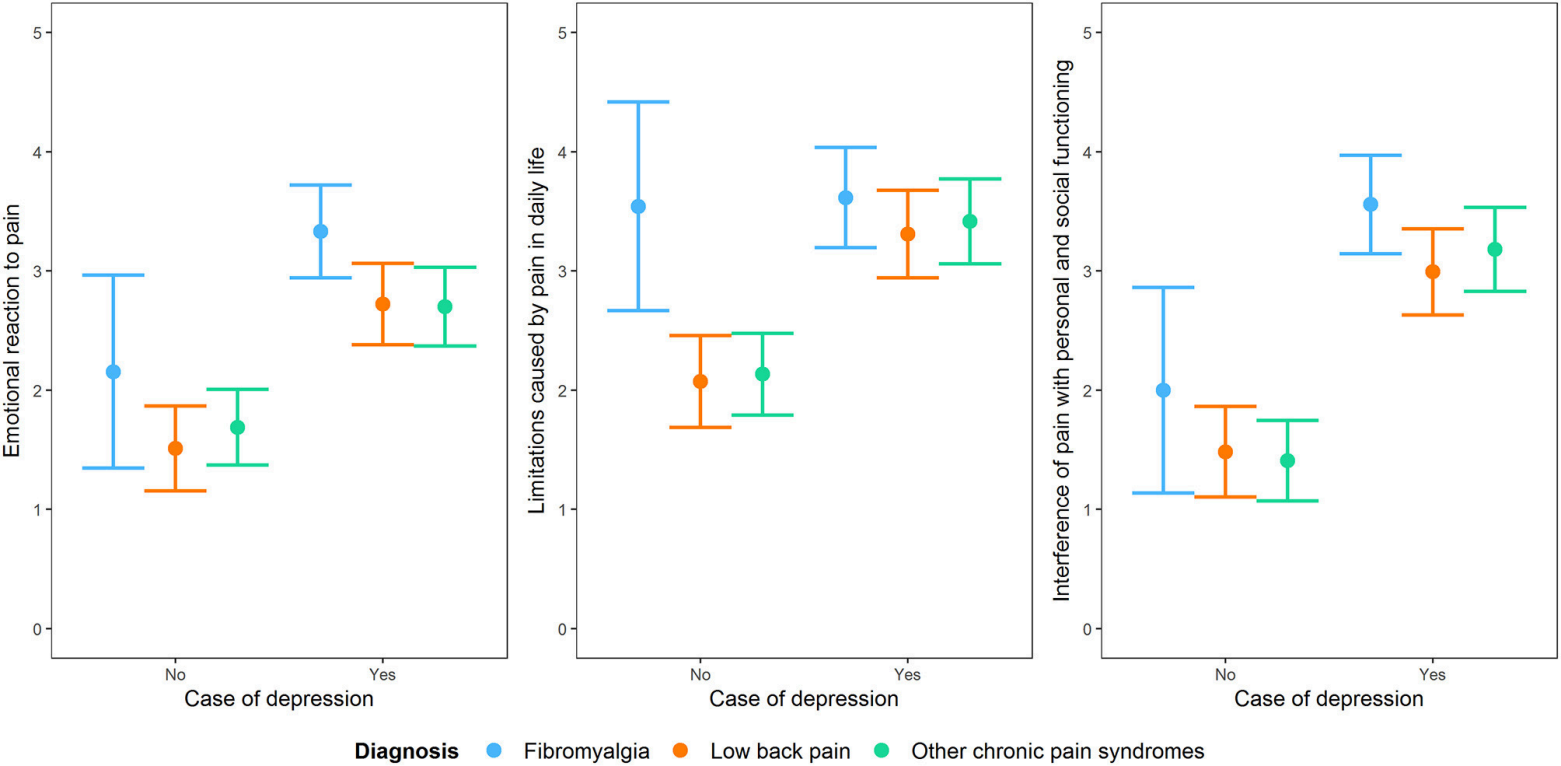
Interaction plot of the impact of being a case of depression at the PHQ-9 on the three dimensions of the BEEP (“emotional reaction to pain,” “limitations caused by pain in daily life,” and “interference with personal and social functioning”) by taking into account diagnostic membership to one of the three conditions of chronic pain syndrome, i.e., fibromyalgia, low back pain and other chronic pain syndromes not including low back pain.

The role of depression remained statistically important when considering the diagnostic membership to a chronic pain syndrome and the interaction between depression and that specific diagnostic membership, with the exception of the subscale “limitations caused by pain in daily life.” For this subscale, diagnostic membership to a chronic pain syndrome was more important than having depression, with participants with fibromyalgia scoring higher than those with the other two conditions (Figure 5).

Since the MDQ had marginal links with the three dimensions of the BEEP, its impact was not explored further.

## Discussion

In this pilot study, the BEEP revealed good reliability both as a global scale and with reference to its three dimensions. CFA proved that the 23 Likert items of the BEEP group fit well in the three expected a-priori dimensions of emotional reaction to pain, limitations caused by pain in daily life, and interference of pain in personal and social functioning. The BEEP also proved able to distinguish among different types of chronic pain syndromes, with participants diagnosed with fibromyalgia scoring higher than the participants diagnosed with low back pain or other chronic pain syndromes on all the dimensions measured by the tool. The levels of depression influence the discriminative capacity of the three dimensions of the BEEP. In particular, being recognized as affected by depression at the PHQ-9 was more important than having a chronic pain syndrome when explaining the distribution of the scores in the subscales “emotional reaction to pain” and “interference with personal and social functioning.” Conversely in the BEEP subscale measuring “limitations caused by pain in daily life,” having fibromyalgia was a stronger predictor of the distribution of the scores in the sample than having depression. This may suggest that patients with fibromyalgia experience greater limitations in their daily lives as a consequence of chronic pain than the patients affected by other forms of chronic pain, and this happens regardless from depression. It should be borne in mind that only two thirds of patients with fibromyalgia filled in the PHQ-9, therefore these findings should be taken with caution and require replication in independent samples.

Another point worthy of consideration is that the bifactor model with three orthogonal factors featured a good fit to the data. Benchmarking indicators proved that this bifactor model is a reasonable description of the data, and it subsumes a global score of general “reaction to pain” in the replies of the participants to the items. This may indicate that the BEEP measures a general reaction to pain principally, which is largely influenced by comorbidity with affective disorders. Nevertheless, we decided to privilege the original a-priori conceptualization of the scale because the correlated three-factor model, too, had a reasonable fit to the data, and because it allows a more articulate profile. As a matter of fact, we agree with Reise et al. in saying that: “structural models of a particular form are specified, estimated, and argued for in order to create (not discover) latent variables that serve as proxies for individual differences on psychological traits” (Bonifay et al., 2016, p. 2). Shortly, if a necessary condition to enter a room is that the door is unlocked, proving that the door is unlocked is conceptually distinct from proving that someone walked through it to enter the room (Borsboom et al., 2004, p. 1,062). In other words, a good fit of a bifactor model does not mean that the data are necessarily described by a single factor with some additional nuances.

The higher scores achieved by the participants with fibromyalgia on all dimensions of the BEEP are probably a reflection of the greater importance of the emotional component in the reaction to pain in these patients. Indeed, levels of depression were associated at a high effect size with the scores on the BEEP, suggesting that depression is a factor that influences the impact of pain in people with chronic pain syndromes; in fact, it explains the distribution of the scores in the sample independently from chronic pain type, with the exception of the dimension of limitations caused by pain in daily life. Overall, the study confirmed the high rate of mood disorders in people diagnosed with chronic pain syndrome. About 50% of the participants who filled in the questionnaires had a score of probable major depression on the PHQ-9, and 7 to 15% were positive for probable bipolar disorder on the MDQ as against 4% in the general population (Carta et al., 2014).

The high prevalence of mood disorders in people with chronic pain syndromes exposes these patients to the risk of suicide (Hooley et al., 2014; Racine, 2017), and probably contributes to a pejorative course of the somatic condition, which might account partially for the high burden in terms of years lived with disability (GBD Disease and Injury Incidence and Prevalence Collaborators, 2017). Patients with fibromyalgia are especially exposed to the higher burden caused by a mood disorder (Carta et al., 2018). A mood disorder might be a predisposing factor for the development of fibromyalgia (Chang et al., 2015). Mood disorders can also contribute to worsening the quality of life in people with fibromyalgia (Sancassiani et al., 2017; Carta et al., 2018). Finally, they may affect the choice of treatment (Carta et al., 2006). Antidepressants are the drug of choice in the treatment of depression and are helpful in the therapy of anxiety. There is also some evidence of their effectiveness in the management of chronic pain, especially neuropathic pain (Mika et al., 2013; Finnerup et al., 2015). However, patients who have a vulnerability to bipolar disorder are also more sensitive to the irritability that can be triggered by antidepressants and may be more prone to switch into mania (Allain et al., 2017; Scott et al., 2017). There is evidence that patients with fibromyalgia get worse under antidepressant treatment (Carta et al., 2013). A similarly poor impact of treatment with antidepressants may be anticipated in patients with other chronic pain syndromes when they have comorbid bipolar disorder.

Within this perspective, the BEEP might be helpful in assessing the impact of pain in patients with chronic pain syndromes. The BEEP assesses three dimensions that may be sensitive to pain in different syndromes, in different ways. Patients with generalized sensitivity to pain, as those with fibromyalgia, may report high scores in all dimensions. On the contrary the patients with more localized pain, like those suffering from a migraine or chronic neck pain, might report more limitations and interference from pain than an emotional reaction to it. This study provided evidence that the levels of depression are highly associated with scores on the three dimensions of BEEP. Therefore, high scores on the BEEP may be considered as an indication that the patient may have a comorbid mood disorder. As a matter of fact an in-depth psychiatric assessment of patients with chronic pain syndromes is always advisable, because of their high comorbidity with a mood disorder. The BEEP may serve the purpose of an entry-level screening tool, to be followed by more appropriate assessment at a secondary stage. Being a multidimensional measure of the impact of pain, the BEEP may be helpful also in evaluating the response over time to the treatment of the patient and may favor the identification of unmet needs in the personal, social and daily functioning of the patients.

Future studies will be necessary to establish the different profiles of responding to the BEEP in generalized vs. localized chronic pain syndromes and in neuropathic vs. non-neuropathic variants (e.g., Dworkin et al., 2007). Indeed, this pilot study is not exempt from limitations. We did not evaluate the convergent and divergent validity of the questionnaire with other measures of pain and with social desirability. Reliability was evaluated as internal consistency only, but we did not evaluate the test-retest stability of the questionnaire, which will be evaluated in future investigations. One of the groups under investigation was a heterogeneous, residual group including patients with disparate chronic pain syndromes. This may have limited the investigation of the discriminative properties of the BEEP. Finally, a minority of the patients did not fill in the questionnaires aimed at investigating comorbidity with affective disorders, thus limiting the conclusions that may be derived from the investigation of the role of depression in the responses to the BEEP. As suggested by a reviewer, another limitation of the study concerns the lack of formal documentation on the process that let to the design of the questionnaire, such as the rationale for including and excluding items, formal testing of discriminant content validity as suggested by Johnston et al. (2014), and so on. It is advisable that future studies on the development of questionnaires keep close track of these steps.

## Author Contributions

AP and MC designed, and wrote the manuscript. SS, FP, MD, MM, FS, FR, and SM revised the manuscript sections. All authors revised the whole paper and approved the final version of the manuscript.

### Conflict of Interest Statement

The authors declare that the research was conducted in the absence of any commercial or financial relationships that could be construed as a potential conflict of interest.
