# Supplementary material for: BEEP—Bodily and Emotional Perception of Pain. A Questionnaire to Measure Reaction to Pain in Chronic Pain Disorders
Source: Front Psychol. 2019 Mar 12;10:480. doi: 10.3389/fpsyg.2019.00480 (PMC6422924; doi:10.3389/fpsyg.2019.00480)
Supplement: Supplementary file 1 [file Data_Sheet_1.PDF]

Appendix

Bodily and Emotional Perception of Pain (BEEP) – English version

[At retest, add first the phrase "Taking into account the last month"]
On a scale from 0 to 5, please specify how intense were the following moods in the occasion you felt the strongest pain:

1.	Irritability<br>(I lose my patience at the slightest thing)	0	1	2	3	4	5
2.	Feeling powerless	0	1	2	3	4	5
3.	Depression<br>(deep sadness with loss of any interest)	0	1	2	3	4	5
4.	Feeling of injustice (why me?)	0	1	2	3	4	5
5.	Pessimism (a negative vision of the future)	0	1	2	3	4	5
6.	Anxiety	0	1	2	3	4	5
7.	Feeling guilty<br>(for example: I feel I am a burden for my family)	0	1	2	3	4	5
8.	Frustration<br>(I can do nothing about it and I am angry at this)	0	1	2	3	4	5
9.	I lack confidence in my abilities and skills	0	1	2	3	4	5
10.	I'm afraid I will not recover	0	1	2	3	4	5
11.	Confusion (I feel my mind is less clear)	0	1	2	3	4	5
12.	I do not recognize myself	0	1	2	3	4	5
13.	I feel I have become older	0	1	2	3	4	5
14.	I feel impaired	0	1	2	3	4	5
15.	I don't feel independent	0	1	2	3	4	5

[At first contact, use this phrase]
On a scale from 0 to 5, during your lifetime, how seriously did have this pain

[At retest, use this phrase]
On a scale from 0 to 5, in the past month, how seriously did have this pain

Limited:

16. Your working performance	0 1 2 3 4 5
17. Your capability to move	0 1 2 3 4 5
18. Your social role	0 1 2 3 4 5
19. Your sports activity	0 1 2 3 4 5

Interfered with:

20. Mood (inner affective-emotional tone, e.g.: I'm more often sad, more often cheerful)	0 1 2 3 4 5
21. Interpersonal relationships	0 1 2 3 4 5
22. Sleep	0 1 2 3 4 5
23. The pleasure of living	0 1 2 3 4 5

Please tick the number that describes the severity of your pain best:

24. On average in the past 24 hours

0 1 2 3 4 5 6 7 8 9 10

25. In this moment

0 1 2 3 4 5 6 7 8 9 10

QUESTIONARIO PERCEZIONE EMOTIVA E CORPOREA DEL DOLORE
(PEC-25) – Italian version

[Se usata al retest, premettere la frase “Considerato l’ultimo mese”]
Su una scala da 0 a 5 indichi con quale <u>intensità</u> ha provato i seguenti <u>stati d'animo</u> nei momenti di maggiore dolore:

1. Irritabilità (perdo la pazienza per un nonnulla)	0	1	2	3	4	5
2. Sentimento di impotenza	0	1	2	3	4	5
3. Depressione (profonda tristezza con perdita di interesse)	0	1	2	3	4	5
4. Senso di ingiustizia (perché io?)	0	1	2	3	4	5
5. Pessimismo (visione negativa del futuro)	0	1	2	3	4	5
6. Ansia	0	1	2	3	4	5
7. Senso di colpa (ad esempio: mi sento un peso per la mia famiglia)	0	1	2	3	4	5
8. Frustrazione (non posso farci niente e mi fa rabbia)	0	1	2	3	4	5
9. Sfiducia nelle mie capacità	0	1	2	3	4	5
10. Paura di non guarire	0	1	2	3	4	5
11. Confusione (mi sento meno lucido)	0	1	2	3	4	5
12. Non riconosco me stesso	0	1	2	3	4	5
13. Mi sento invecchiato	0	1	2	3	4	5
14. Mi sento menomato	0	1	2	3	4	5
15. Non mi sento indipendente	0	1	2	3	4	5

[Se usata al primo contatto, usare questa espressione]:
Su una scala da 0 a 5 con quale gravità, nel corso della sua vita, la sindrome dolorosa ha

[Se usata al retest, usare questa espressione]:
Su una scala da 0 a 5 con quale gravità, nel corso dell'ultimo mese, la sindrome dolorosa ha

Limitato:

16. La sua prestazione lavorativa	0	1	2	3	4	5
17. La sua capacità di movimento	0	1	2	3	4	5
18. Il suo ruolo sociale	0	1	2	3	4	5
19. La sua attività sportiva	0	1	2	3	4	5

Interferito con:

20. L'umore (tonalità affettivo-emotiva interna, ad esempio: più spesso triste, più spesso allegro)	0	1	2	3	4	5
21. Le relazioni interpersonali	0	1	2	3	4	5
22. Il sonno	0	1	2	3	4	5
23. Il piacere di vivere	0	1	2	3	4	5

Indichi il numero che meglio descrive l'intensità del suo dolore:

24. In media nelle ultime 24 ore

0 1 2 3 4 5 6 7 8 9 10

25. In questo momento

0 1 2 3 4 5 6 7 8 9 10
